# Frequency and Characteristics of Postoperative Neuropathy in Individuals on Gender-Affirming Hormone Therapy Undergoing Gender Affirmation Surgery: A Retrospective Cohort Study

**DOI:** 10.7759/cureus.47988

**Published:** 2023-10-30

**Authors:** Brandon M Togioka, Kevin A Harriman, Shangyuan Ye, Jens Berli

**Affiliations:** 1 Anesthesiology and Perioperative Medicine, Oregon Health & Science University, Portland, USA; 2 Anesthesiology, Riverside Community Hospital, Riverside, USA; 3 Biostatistics, Oregon Health & Science University, Portland, USA; 4 Plastic Surgery, Oregon Health & Science University, Portland, USA

**Keywords:** gender-affirming hormone therapy, iatrogenic nerve injury, postoperative neuropathy, gender-affirming care, gender-affirming surgery, transgender sex reassignment surgery, transgender surgery

## Abstract

Introduction

Gender affirmation surgery includes procedures of the face, larynx, chest, reproductive system, external genitalia, and adipose tissue performed to ameliorate incongruence between gender identity and phenotype. The annual number of gender affirmation surgeries performed in the United States has increased significantly. There have been no investigations into the frequency of peripheral neuropathy after gender affirmation surgery, which is an important topic, given transgender individuals are at increased risk for delaying necessary medical care. After appreciating a number of cases of postoperative neuropathy in our clinical practice, we hypothesized that gender affirmation surgery is a high-risk procedure for postoperative neuropathy.

Methods

We conducted a one-year, monocentric, retrospective cohort study utilizing clinical data of individuals on gender-affirming hormone therapy undergoing gender-affirmation surgery under general anesthesia. The study included transgender women, assigned male at birth, receiving antiandrogen, progesterone, or estrogen therapy (target range plasma estradiol concentration 90-200 pg/ml) and transgender men, assigned female at birth, receiving antiestrogen or testosterone therapy (target range plasma testosterone concentration 320-1000 ng/dl). The primary objective was to estimate the incidence of postoperative peripheral neuropathy, defined as new numbness, paresthesia, neuropathic pain, or muscle weakness occurring in a peripheral innervation territory. Secondary objectives were to summarize the clinical presentation of neuropathy and investigate for associations between procedure characteristics and neuropathy.

Results

We identified nine cases of postoperative peripheral nerve injury in 232 consecutive gender affirmation procedures establishing an incidence of 3.9%. All injuries were associated with surgery longer than six hours and were characterized by sensory deficits including numbness 89% (8/9) and tingling 56% (5/9), which were diagnosed by postoperative day one.

Conclusions

Our results suggest that gender affirmation surgery is a high-risk procedure for postoperative neuropathy, with an incidence similar to other high-risk procedures, and an incidence that is higher than the general surgical population. However, given this has not been previously reported and our study includes a heterogenous population from a single institution, our results should be considered hypothesis generating. Additional studies that include multiple institutions are needed to confirm our findings and identify modifiable risk factors for postoperative neuropathy.

## Introduction

Gender affirmation surgery (GAS) refers to procedures that aim to ameliorate incongruence between gender identity and phenotype, which may cause distress referred to as gender dysphoria [1]. The modern era of GAS began in 1931 with a vaginoplasty performed in the German Institute of Sexual Science, under the direction of Dr. Hirschfeld [2]. GAS now includes procedures of the face (frontal bone reduction, rhinoplasty, jawline contouring), larynx (laryngeal chondroplasty), chest (mastectomy and breast augmentation), reproductive system (gonadectomy, hysterectomy), external genitalia (metoidioplasty, phalloplasty, vulvoplasty, vaginoplasty) and adipose tissue (liposuction, fat grafting). The prevalence of GAS has increased significantly since 2010, a finding thought to be partially attributable to the prohibition of gender-based discrimination by section 1557 of the Affordable Care Act [3].

In 2019, we reviewed three cases of post-GAS neuropathy amongst individuals on gender-affirming hormone therapy (GAHT) within a single quality review committee meeting. This finding served as the impetus for our study. We hypothesized that GAS is a high-risk procedure for postoperative neuropathy with an incidence greater than the general surgical population. Given Oregon Health and Science University has one of the largest Transgender Health Programs in the United States, it was felt that our population size may be large enough to provide meaningful knowledge. There have been no investigations into the prevalence of neuropathy after GAS, which is an important topic of investigation, given transgender individuals are at increased risk for delaying necessary medical care and postoperative neuropathy is associated with worse quality of life and at times, permanent disability [3-6].

The primary objective of this retrospective, observational study was to estimate the incidence of neuropathy after GAS performed on individuals receiving GAHT. Secondary objectives were to summarize the clinical presentation of neuropathy and investigate for associations between procedural characteristics and postoperative neuropathy.

## Materials and methods

Study design and setting

We conducted a one-year (2019) retrospective, observational cohort study utilizing electronic health records from a single academic institution, Oregon Health & Science University (OHSU) in Portland, Oregon, United States. The study protocol, including inclusion and exclusion criteria, outcomes, and plan for data analysis was written and approved by the Oregon Health & Science University Institutional Review Board (STUDY 20625) on January 14, 2020, before accessing the medical records data. The requirement for written informed consent was waived by the institutional review board. As an observational study, the anesthetic practice was not standardized; the method of blood pressure monitoring, patient positioning, pressure point padding, transfusion threshold, neuromuscular blockade and antagonism, method of neuromuscular monitoring, treatment of hypotension, and patient position checks were at the discretion of the anesthesia team and in accordance with routine anesthesia practice within the study site.

Search strategy 

GAS were identified via a search of our electronic health records (Epic Systems Corporation, Verona, Wisconsin, United States). The initial search was intentionally expansive to increase sensitivity; all procedures performed by surgeons in our gender affirmation program during the study period were assessed. For each procedure, one investigator collected de-identified data on patient demographics, patient characteristics, preoperative comorbidities, procedure characteristics, and laboratory values. Assessment of neuropathy was completed by two independent investigators, who classified cases as neuropathy present or neuropathy absent, and extracted neuropathy characteristics. An a priori plan was created to have disagreements resolved by group discussion. This manuscript adheres to the applicable Strengthening The Reporting of Observational Studies in Epidemiology (STROBE) guidelines for cohort studies [7].

Study population

The study population included the American Society of Anesthesiologists (ASA) physical status I-III transgender individuals who were receiving GAHT and undergoing elective GAS involving the chest, face, or genitals under general endotracheal anesthesia. The study included transgender women, assigned male at birth (AMAB), receiving antiandrogen, progesterone, or estrogen therapy (target range plasma estradiol concentration 90-200 pg/ml) and transgender men, assigned female at birth (AFAB), receiving antiestrogen or testosterone therapy (target range plasma testosterone concentration 320-1000 ng/dl) [8]. The study was restricted to individuals on GAHT to create a more homogenous study population and because of the very high prevalence of GAHT (> 99%) among individuals having GAS at the study site. Individuals aged 18 to 90 years who underwent surgery between January 1, 2019, and December 31, 2019, were eligible. To avoid carryover effects for individuals undergoing more than one gender affirmation procedure, only the first procedure (primary GAS) was included. Individuals were excluded if they possessed preoperative neurologic impairment, numbness, or paresthesia; the objective was to estimate the incidence of postoperative neuropathy amongst individuals without pre-existing injury.

Outcomes

The primary outcome variable, postoperative peripheral neuropathy, was defined as numbness, paresthesia (tingling or “pins and needles” sensation), neuropathic pain (shooting, burning, dysesthesia, or allodynia), or muscle weakness occurring in a peripheral innervation territory and diagnosed during the index hospitalization [9,10]. No restrictions were placed upon the diagnosis of neuropathy with respect to a minimum duration of symptoms; temporary neuropathy was considered significant. Numbness, paresthesia, or pain that was clearly attributable to the surgical procedure based upon location, such as sensory changes occurring at a graft donor site, were excluded. Vague symptoms not corresponding to a peripheral innervation territory or to the central nervous system topographic map were excluded. 

Secondary outcome variables characterized the nerve injury and included the location of injury (upper versus lower extremity), type of deficit (sensory, motor, both), the nerve territory within which symptoms were distributed, terms used to describe the neuropathy, time of diagnosis, and whether neuropathy was still present upon discharge.

Patient and procedure variables

Patient demographic data were analyzed, including gender identity or non-binary status, age, race and ethnicity, weight, body mass index, and ASA classification. Patient characteristics assessed included obstructive sleep apnea, asthma or chronic obstructive pulmonary disease requiring chronic bronchodilator therapy, current tobacco use, hypertension requiring medical therapy, coronary artery disease, cerebrovascular disease, peripheral vascular disease, preoperative creatinine, anemia, preoperative hematocrit, diabetes mellitus requiring oral or insulin therapy, thyroid disease requiring medical therapy, history of alcohol use disorder, and history of illicit drug abuse. Procedure characteristics assessed included procedure type and location, duration, estimated blood loss (EBL), method of blood pressure measurement (arterial line versus non-invasive measurement), frequency of non-invasive blood pressure measurement, patient position, number of pressure point checks during anesthesia time, hypotension (defined as > 20 minutes of anesthesia time with mean arterial pressure < 65 mmHg), hyperglycemia (glucose > 180 mg/dl), hypoglycemia (glucose < 70 mg/dl), and provision of peripheral or neuraxial nerve block.

Statistical analysis

As a descriptive study, sample size calculations were not performed. Descriptive statistics were used to summarize the cohort’s baseline demographic data, patient characteristics, procedure characteristics, laboratory values, and postoperative neuropathy characteristics. Continuous variables were summarized with measures of central tendency (mean) and dispersion (standard deviation). Categorical variables were summarized with frequency (percentage). 

To facilitate inclusion of continuous variables in a risk analysis index, certain patient and procedural characteristics were transformed into the following ordinal categorical variables: age was dichotomized as < 30 and ≥ 30 years, body mass index (BMI) was dichotomized using the threshold for morbid obesity (< 40 kg/m^2^ and ≥ 40 kg/m^2^), procedure duration was dichotomized as < 6 hours and ≥ 6 hours, and EBL was dichotomized as < 250 ml and ≥ 250 ml. Given that the electronic health records prompt anesthesia providers to document pressure point assessment at the beginning of surgery and every three hours thereafter, and a high proportion of GAS is of duration ≥ 6 hours, ≥ 4 pressure point checks were considered significant. Race and ethnicity were classified as “White”, “Hispanic”, “Asian”, “Other”, and “Declined to Answer.” Gender affirmation procedures were classified as face, chest, and genital. 

Point estimates for the frequency of neuropathy were determined by dividing the number of individuals with postoperative neuropathy by the total number of individuals within a cohort. We investigated the association between neuropathy and procedural characteristics (those with biological plausibility and those previously associated with postoperative neuropathy) with odds ratios (OR) and corresponding 95% confidence intervals (CI) calculated using Fisher’s exact method. All analyses were conducted using R (version 4.1.1; R Foundation for Statistical Computing, Vienna, Austria). 

## Results

Subject flow

**Figure 1:**
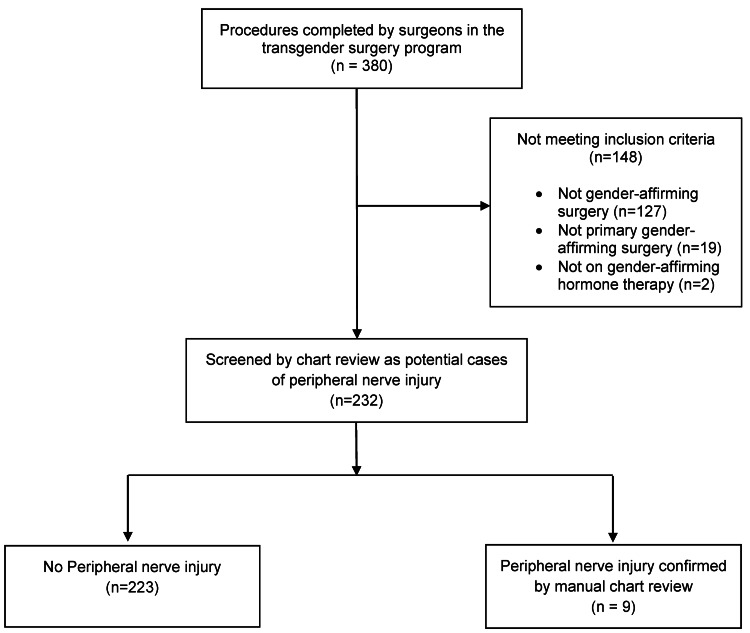
Flow chart showing case inclusion and peripheral nerve injury identification according to the STROBE statement. STROBE, Strengthening The Reporting of Observational Studies in Epidemiology

A total of 380 procedures requiring general anesthesia were completed by five surgeons in the Transgender Health Program in 2019, and 253 of these procedures were gender-affirming. Eighteen patients underwent multiple gender-affirmation procedures (one had three procedures and 17 had two procedures); to avoid a carryover effect, only the first procedure (primary GAS) was included in the data set, excluding 19 cases. A sample of 232 unique cases were screened for postoperative neuropathy (Figure 1). Intraoperative care for these 232 cases involved 63 different anesthesia attending physicians.

Study population baseline characteristics

Patient demographics are presented in Table 1. The mean age of all individuals undergoing GAS was 38.2 years and 59% (138/232) were transitioning from male to female. Most individuals were White (78% = 182/232), not morbidly obese (96% = 215/225), and possessed a low burden of medical co-morbidities (91% = 212/232 were ASA physical status one or two).

**Table 1:** Patient demographics stratified by presence of neuropathy ASA, American Society of Anesthesiologists; BMI, body mass index; kg, kilogram; m, meter; SD, standard deviation.

Demographics	New Postoperative Peripheral Neuropathy (n=9)	No Neuropathy (n=223)
Gender; n (%)		
Male to Female	6 (67%)	132 (59%)
Female to Male	3 (33%)	91 (41%)
Age, years; mean (SD)	40.8 (15.6)	38.1 (13.4)
Age < 30 years, n (%)	2 (22%)	72 (32%)
Age ≥ 30 years, n (%)	7 (78%)	151 (68%)
Race and ethnicity; n (%)		
White	6 (67%)	176 (79%)
Hispanic	0 (0%)	14 (6%)
Asian	1 (11%)	13 (6%)
Other	1 (11%)	8 (4%)
Declined to answer	1 (11%)	12 (5%)
Weight, kg; mean (SD)	86.6 (22.1), n=9	82.8 (21.6), n =217
BMI, kg m^-2^, mean (SD)	28.1 (6.5), n=8	27.9 (6.7), n=217
BMI < 40 kg m^-2^, n (%)	8 (100%)	207 (95%)
BMI ≥ 40 kg m^-2^, n (%)	0 (0%)	10 (5%)
ASA physical status; n (%)		
1	4 (44%)	64 (29%)
2	5 (56%)	139 (62%)
3	0 (0%)	19 (9%)
4	0 (0%)	1 (<1%)

Patient characteristics are included in Table 2. There was a lower prevalence of current tobacco use (0% versus 7%), past alcohol use disorder (33% versus 44%), past illicit drug use (22% versus 40%), hypertension (0% versus 13%), and diabetes mellitus (0% versus 7%) amongst individuals who developed postoperative neuropathy. 

**Table 2:** Patient characteristics stratified by presence of neuropathy ^a^ Anemia was defined as hematocrit < 36% COPD, chronic obstructive pulmonary disease; dl, deciliter; mg, milligram; SD, standard deviation.

Patient Characteristics	New Postoperative Peripheral Neuropathy (n=9)	No Neuropathy (n=223)
Obstructive Sleep Apnea; n (%)	0 (0%)	12 (5%)
Asthma; n (%)	2 (22%)	19 (9%)
COPD; n (%)	0 (0%)	1 (<1%)
Current Tobacco Use; n (%)	0 (0%)	16 (7%)
Hypertension; n (%)	0 (0%)	30 (13%)
Coronary Artery Disease; n (%)	0 (0%)	1 (<1%)
Cerebrovascular Disease; n (%)	0 (0%)	0 (0%)
Peripheral Vascular Disease; n (%)	0 (0%)	0 (0%)
Creatinine, mg/dl; mean (SD)	0.82 (0.11), n=6	0.87 (0.16), n=94
Anemia; n (%) ^a^	0 (0%)	2 (<1%)
Starting Hematocrit, %; mean (SD)	42.3% (4%), n=6	42.4% (4%), n=105
Diabetes Mellitus; n (%)	0 (0%)	16 (7%)
Hypothyroid; n (%)	0 (0%)	10 (4%)
Alcohol Use Disorder History; n (%)	3 (33%)	99 (44%)
Illicit Drug History; n (%)	2 (22%)	89 (40%)

Procedure characteristics

Procedure characteristics are presented in Table 3. Procedure duration ≥ 6 hours (OR=$\infty$; 95%CI, 2.11-$\infty$) and ≥ 4 pressure points assessments (OR=$\infty$; 95%CI, 2.15-$\infty$) were associated with postoperative neuropathy. There was a trend towards greater blood loss in the postoperative neuropathy cohort than the cohort without neuropathy, mean EBL 289 ml (SD 127) versus 185 ml (SD 172). Cases of postoperative neuropathy had 2.3 times the odds of being associated with EBL ≥ 250 ml (OR=2.31; 95%CI, 0.48-12.02), but this difference did not reach statistical significance. Genital surgery (compared to face and chest), non-invasive blood pressure measurement at three-minute intervals (compared to the measurement at five-minute intervals), lithotomy position (compared to supine position), hypotension, and hyperglycemia were not associated with postoperative neuropathy. No patients experienced hypoglycemia, and there were no peripheral or neuraxial nerve blocks performed.

**Table 3:** Procedure characteristics stratified by presence of neuropathy ^a^ The odds ratio for procedure location was obtained via Fisher’s exact method comparing genitalia with the grouping of cranium and thoracic. ^b^ The odds ratio for blood pressure monitoring was obtained with Fisher’s exact method comparing three-minute and five-minute interval checks, cases that primarily utilized intra-arterial pressure measurement were excluded. dl, deciliter; EBL, estimated blood loss; MAP, mean arterial pressure; mg, milligram; ml, milliliter; mm Hg, millimeters of mercury; SD, standard deviation.

Procedure Characteristic	New Postoperative Neuropathy (n=9)	No Neuropathy (n=223)	Odds Ratio (95% CI)
Procedure location; n (%)			
Cranium	2 (22%)	14 (6%)	---
Thoracic	0 (0%)	30 (14%)	---
Genital ^a^	7 (78%)	179 (80%)	0.86 (0.16, 8.78)
Procedure duration, hour; mean (SD)	7.7 (1.4)	4.9 (2.8)	
Duration ≥ 6 hours	9 (100%)	106 (48%)	$\infty$ (2.11, $\infty$)
EBL, ml; mean (SD)	289 (127)	185 (172)	
EBL ≥ 250 ml	5 (56%)	78 (35%)	2.31 (0.48, 12.02)
Blood Pressure Monitoring; n (%)			
Intra-arterial pressure measurement	0 (0%)	13 (6%)	---
Non-invasive cuff measurement at the three-minute interval ^b^	3 (33%)	125 (56%)	0.34 (0.05, 1.65)
Non-invasive cuff measurement at the five-minute interval	6 (67%)	85 (38%)	---
Patient Position; n (%)			
Supine	3 (33%)	91 (41%)	---
Lithotomy	6 (67%)	132 (59%)	1.38 (0.29, 8.72)
Number of Pressure Point Checks; n (%)			
≥ 4	9 (100%)	105 (47%)	$\infty$ (2.15, $\infty$)
Hypotension; n (%)			
MAP < 65 mm Hg longer than 20 min	0 (0%)	6 (3%)	0 (0, 23.71)
Hyperglycemia; n (%)			
Glucose > 180 mg/dl	0 (0%)	7 (3%)	0 (0, 19.43)

Primary and secondary endpoints

The primary endpoint, incidence of postoperative neuropathy, and the secondary endpoints, which characterized the clinical presentation of neuropathy, are summarized in Table 4. The one-year frequency of postoperative peripheral neuropathy after GAS was nine of 232 cases or 3.9%. Among postoperative neuropathies, 56% (5/9) occurred in the upper extremity (three in supine and two in lithotomy position), compared to 44% (4/9) in the lower extremity (all four in lithotomy position). All postoperative neuropathies were associated with sensory deficits. The most common symptoms were numbness (89%, 8/9) and tingling (56%, 5/9). Neuropathy was diagnosed on the night of surgery or on postoperative day one, and in 78% (7/9) of cases, it was still present upon discharge. Due to the small number of identified postoperative neuropathy cases, a risk analysis index could not be created. 

**Table 4:** Characteristics of identified postoperative neuropathies

Characteristic	Postoperative Neuropathy (n=9)
Location; n (%)	
Upper extremity	5 (56%)
Lower extremity	4 (44%)
Type of deficit; n (%)	
Sensory	9 (100%)
Motor	0 (0%)
Distribution of symptoms, nerve territory; n (%)	
Brachial plexus	2 (22%)
Sciatic	2 (22%)
Lateral femoral cutaneous	1 (11%)
Common peroneal	1 (11%)
Radial	1 (11%)
Median	1 (11%)
Ulnar	1 (11%)
Paresthesia and pain descriptors; n (%)	
Numbness	8 (89%)
Tingling	5 (56%)
Shooting pain	2 (22%)
Burning pain	1 (11%)
Time of diagnosis, postoperative day; n (%)	
0	2 (22%)
1	6 (67%)
Uncertain	1 (11%)
Neuropathy present on discharge; n (%)	7 (78%)

## Discussion

Key results and interpretation

In this small retrospective study, one postoperative neuropathy case occurred for every 26 gender affirmation procedures (3.9%). Indeed, this represents a notable risk to transgender individuals. This finding suggests that GAS is associated with a higher frequency of postoperative neuropathy than the general surgical population. For comparison, two large single-institution retrospective studies and one systematic review found that the frequency of postoperative neuropathy within a broad surgical population is between 0.007% and 0.03% [11-13].

In our study, two covariates found to be associated with postoperative neuropathy were longer procedure duration and an increased number of pressure point assessments. These covariates are associated. Our finding that longer-duration procedures are associated with postoperative neuropathy is consistent with prior studies [4,14,15]. The average duration of procedures included in our study was five hours. Our observed frequency of postoperative neuropathy (3.9%) is comparable to other surgeries of long duration (generally greater than four hours) including laparoscopic surgery (1.2-4.7%) [15,16], robotic-assisted laparoscopic proctectomy (1.3-6.6%) [14,17], and cardiac surgery (6.1%) [18].

The frequency of postoperative neuropathy found in our study positions GAS among the highest-risk surgical categories. Other categories of surgery associated with a high risk for postoperative neuropathy include long-duration laparoscopic surgery (1.2-4.7%) [15,16], robotic-assisted laparoscopic prostatectomy (1.3-6.6%) [14,17], total knee arthroplasty (2.2%) [19], shoulder arthroplasty (4.3%) [20], and major cardiac surgery (6.1%) [18]. Direct comparison between our findings and the frequency of neuropathy reported in these studies is challenging and subject to detection bias; the literature includes heterogeneous definitions that vary with respect to the minimum duration of symptoms required for a diagnosis and the method of neuropathy verification (clinical signs and symptoms, nerve conduction studies, and electromyography).

Postoperative lower extremity neuropathy is a well-known complication of the lithotomy surgical position [4,17]. In the current study, the frequency of lower extremity neuropathy amongst patients in the lithotomy position was four of 138 or 2.9%. All cases were associated with a procedure duration greater than six hours. Our observed frequency is comparable to studies that assess long-duration lithotomy positioning including surgery involving the urinary tract, reproductive organs, and gastrointestinal tract (1.5%) [6], and robotic-assisted proctectomy (5.1-6.6%) [17,21].

The pathophysiology of neuropathy after GAS is unknown and likely multifactorial. One unique factor may be GAHT. Adjusting sex hormone concentrations to be outside the physiologic range has been associated with increased peripheral nerve vulnerability to injury [22,23]. The high incidence of carpal tunnel in pregnancy, 31-62% when clinically diagnosed and 7-43% with electrodiagnostic studies, has been attributed to elevated progesterone levels causing median nerve hypersensitivity and compression [24]. Physiologic levels of androgen and estrogen promote trophic changes and motor neuron regeneration following injury [22,25-28]; whereas antiandrogen and antiestrogen agents abolish this effect [22,23]. Lastly, exogenous androgen administration has been shown to accelerate facial nerve regeneration in male hamsters, but not females [29]. Accordingly, there may be a biological mechanism by which anti-androgen and GAHT, administered to individuals in the present study, may have sensitized peripheral nerves to intraoperative compression, stretch, and ischemic injury. Another possible etiology in AFAB individuals on testosterone may be attributed to an increase in muscle mass relative to the fascial compartments. These hypotheses could not be tested since the incidence of GAHT in our Transgender Health Program is too high to create a non-exposed cohort for comparison.

The long-term clinical significance of these postoperative neuropathies is uncertain. Due to the retrospective nature of our study, we were unable to assess the duration of symptoms, or whether peripheral nerve deficits were associated with lasting disability. Among studies that conducted follow-ups on neuropathy outcomes, measures and results were variable but the majority of injuries resolved within one year [4-6,14,17,30]. Additional research is needed to determine the long-term effect of neuropathy after GAS on quality of life and daily functioning.

Strengths, limitations, and generalizability

This study has a number of strengths. It is the first known investigation of the incidence of postoperative neuropathy after GAS. It included an expansive electronic health record search that is unlikely to have missed GAS, the use of clinical instead of administrative data, and intensive data mining that included a review of all inpatient and outpatient notes instead of utilizing a database search. Investigations that rely upon database entries and administrative data are likely to miss shorter duration and lower severity neuropathies.

Our analysis has several limitations inherent to retrospective cohort study design including the use of clinical data not intended for research purposes, lack of consistency with respect to measurement of covariates and outcomes due to the involvement of multiple medical providers in subject care, possible misclassification bias due to missed diagnosis or missed documentation of postoperative neuropathy, and the potential for unrecognized confounders. Estimates for ulnar and lower extremity neuropathy, well-established positioning complications, are lower in retrospective (0.04% and 0.03%, respectively) than in prospective studies (0.47% and 1.51%, respectively) [4-6,30]. Accordingly, our retrospective chart review may underestimate the true incidence of postoperative neuropathy.

The small sample size and low quantity of neuropathy events decreased statistical power to detect an association between covariates and postoperative neuropathy. Additionally, the incidence of postoperative neuropathy was compared with a historical [11-13], not a concurrent control group. The low incidence of postoperative neuropathy did not allow us to focus on a single procedure, stratify by type of GAHT, or assess for a GAHT dose-response relationship. To address these limitations, we suggest future studies include multiple institutions to determine if our results are a site-specific quality finding or indeed generalizable. 

External validity is limited because the study population included a high proportion of Caucasian subjects, it was conducted at a single academic institution, and GAS was performed by five surgeons.

## Conclusions

Peripheral neuropathy after GAS has been poorly investigated. In a descriptive retrospective cohort study of 232 consecutive patients on GAHT undergoing GAS, the incidence of postoperative neuropathy was 3.9%. Procedure duration greater than six hours was associated with postoperative neuropathy and all cases of neuropathy were characterized by sensory deficits including numbness (89%) and tingling (56%).

Our observed incidence (3.9%) of postoperative neuropathy is comparable to the frequency of neuropathy after long-duration laparoscopic surgery, robotic-assisted laparoscopic prostatectomy, total knee arthroplasty, shoulder arthroplasty, and major cardiac surgery. This finding is consistent with our study hypothesis that GAS represents a high-risk procedure for postoperative neuropathy. Given that GAHT has not been previously associated with neurologic morbidity and our study includes a heterogeneous population from a single institution, our findings should be considered hypothesis-generating. Our exploratory results indicate the need for additional research within a larger cohort to confirm our findings and to identify modifiable risk factors for individuals undergoing GAS.
